# Medical Society of Erie County

**Published:** 1890-07

**Authors:** William H. Thornton

**Affiliations:** Secretary


### Society Proceedings.

### MEDICAL SOCIETY OF ERIE COUNTY.

Reported by WILLIAM H. THORNTON, M. D., *Secretary*.

THE regular semi-annual meeting of the society was held at the rooms of the Young Men's Christian Association, Tuesday, June 11, 1890.

The meeting was called to order at 11 o'clock A. M., by the President, Dr. G. W. McPHERSON.

The minutes of the previous meeting were read and approved.

The committee on membership reported in favor of the election of the following applicants:

Drs. E. E. Briggs, F. M. Gipple, Allen A. Jones, Wm. C. Krauss, R. E. Moss, M. Retel, Emile Schroeder, Hugo Schmidt, Otto Thoma, and J. C. Thompson.

Upon motion of Dr. J. D. HILL the report of the committee was adopted.

The committee on membership reported the following applications:

Drs. L. B. Dorr, B. S. Bourne, Henry Mulford, E. A. Forsyth, H. Y. Grant, C. O. Chester, C. T. Wolsey, A. F. Andrews.

Upon motion of Dr. EDWARD STORCK it was voted that the secretary send notice to all those who have been elected to-day of their membership, and send them a copy of the by-laws and constitution; also to those who have been elected recently and who have not received such notice.

The Board of Censors presented their report, as follows:

*To the Medical Society of Erie County:*

The Board of Censors, at a former meeting, were directed to bring a test case before a proper tribunal, in order to prove whether the law of 1828, embodied in the statutes of this State, by which membership in a legal medical society is made obligatory upon physicians and surgeons practicing in the county, is still in force. Thereupon your Board engaged legal counsel. Mr. William B. Hoyt furnished a written opinion, which was submitted to the society at a former meeting. According to this opinion the law cited is still in force. Any physician or surgeon who neglects to apply for membership in a legal medical society of the county in which he practises for sixty days, after proper notification by the president of such society to do so, forfeits his license, and if he still continues to practise he makes himself liable to the penalties imposed upon those that practice without license. The Board had notices served by the president upon a number of practitioners in this county, not members of a county medical society, who are regular graduates of legal medical schools, but are doing an advertising business, in violation of medical ethics and by-laws of medical societies. Some of these physicians have made application for membership in this society. Your Board, therefore, cannot commence legal proceedings against those physicians. According to the laws of the State they are entitled to membership in a legal medical society if they are in possession of a diploma or license legally granted, and should be admitted. After they have become members of the society, if they still continue to practice in violation of medical ethics and the by-laws, they then can be disciplined and expelled from the society, when the diploma or license they hold would become null and void. Such a case has been before the Board at its last meeting. Dr. Hugh J. Linn was served with a notice by the President, as he was practising in this county, not being a member of a society in the county. He claimed the right to practise, on the ground that he was a member of the Medical Society of the County of New York. He was since expelled from that society for practising in violation of its by-laws and medical ethics. Your Board will serve notice upon him to desist forthwith from practising medicine until he has complied with the laws of the State, or legal proceedings would be commenced against him. Your Board also have decided to prosecute others who have not made application for membership, as prescribed by law, as soon as the society will take action upon the application now in the hands of the committee on membership.

The following named persons have been reported to the Board as practising medicine in this county in violation of the laws of the State, not having either license or diploma authorizing them to do so:

Frederick Simon, Spring street, Buffalo.
Rudolph Waldvogel, Jefferson street, Buffalo.
Mrs. Bronson, Willink, Aurora.
Sarah A. Woodruff, East Aurora.

These cases will be given to the district attorney, to be brought before the grand jury at its next session, if sufficient evidence will be furnished by the plaintiffs.

There are several druggists in this city who are doing considerable prescription business over the counter, and cases have been reported where serious results have been the consequences. The Board will proceed against them as soon as we have sufficient evidence to convict.

The Board of Censors,
E. Storck,
P. W. Van Peyma,
Henry Lapp,
Jos. L. Haberstro.

Upon motion of Dr. W. W. Potter, it was voted that the report of the Board of Censors be received and an order drawn upon the treasurer for the amount, $50.00, asked for by the board.

Dr. Rochester stated that there were no applications before the committee on membership which had not been acted upon. Thereupon the following clause was stricken from the report of the Board of Censors, as read : " But our committee on membership has, until now, failed to act upon their applications."

Upon motion of Dr. W. W. POTTER it was voted that the report of the Board of Censors be adopted, and that the society endorse the proposed action of the Board, as set forth in their report, against the several individuals named therein.

Dr. WYCKOFF moved that the names of those who have applied for membership and who have not been admitted be reported to the society, and those who have presented diplomas be re-committed to the committee for further consideration.

Dr. STORCK moved to amend that the committee on membership be hereby directed to report in favor of the admission of all applicants who are regular graduates of a legal medical school, if there are no serious objections to them in other respects sufficient, in the opinion of the committee, to warrant a report against them, the objections if any being reported back to the society.

The amendment was carried.

The original motion, as amended, was carried.

The secretary read letters from Assemblymen Sheehan and Andrus, and Congressman Farquhar.

Upon motion of Dr. STORCK the letters were received and filed.

Dr. BRIGGS, from the committee, presented the following memorial upon the death of Dr. A. J. Brooks :

### MEMORIAL OF DR. A. J. BROOKS,

Late of Marilla, Erie County, N. Y.

Andrew J. Brooks was born in the town of Concord, Erie county, N. Y., August 5, 1832, and died at his home in Marilla, N. Y., April 16, 1890, aged 57 years.

Doctor Brooks was the son of John and Lydia Brooks. He received his early education in the district schools of the neighborhood where he was born, afterward attended the academies at Aurora and Springville, and finally graduated from the Genesee Wesleyan Seminary at Lima, N. Y. Shortly afterward he went to the Albany Medical College, from which institution he graduated in 1860, and immediately thereafter located at Marilla, N. Y., where he successfully practised his chosen profession, until stricken down with the illness which caused his death. Dr. Brooks, at the time of his death, was the health officer of his town, having held the office from the time the board of health was organized, in 1883, and was always an efficient and reliable officer. His family who survive him are his wife and six children. The eldest son, Ralph, is now a student of medicine in the medical department of the University of Buffalo.

The doctor's occupation for thirty years was the *practice of medicine*, and that only. It was enough for him to be a doctor, and he never turned aside to seek for political preferment or to gain riches in any other pursuit. His life was the trying one of a country doctor, yet he never complained of his lot, but kept straight on in his work to the end. His death is a great loss to the community in which he lived, to the church in which he was a faithful worker, and to the profession, of which he was an earnest, faithful member.

Your committee would recommend that this sketch be spread on the minutes of this society.

A. H. BRIGGS,

E. T. DORLAND,

M. B. SEARLES,

*Committee.*

Dr. HAUENSTEIN presented the following memorial upon the late Dr. A. Devening:

### MEMORIAL OF DR. DANIEL DEVENING,

Late of Buffalo, N. Y.

By JOHN HAUENSTEIN, M. D.

At the advanced age of seventy-nine years, Dr. Daniel Devening departed this life on March the 5th, 1890. He was graduated at the medical department of the University of New York in 1846, and in the same year began the practice of medicine in this city, in the pursuit of which he was engaged up to the time of his death. Although of late years somewhat retired, and therefore not so generally known by the younger members of the profession, he was so much better known and appreciated by the older members, very few of whom now remain with us.

He was an estimable man, a devoted student, and a conscientious and skilful physician; courteous in his bearing and intercourse with his colleagues, commanding the respect of all of those with whom he came in contact in his professional as well as in every-day life.

He at one time took an active part in military affairs, and acquitted himself with honor as an officer.

In 1855 and '56 he represented his district in the assembly of the State legislature, and in 1858 was a member and chairman of the Common Council. From all these positions he retired with the grateful remembrance of his constituents, after having performed his duties faithfully and honorably.

This society desires to express its sincere and respectful sympathy to the family and friends of the deceased.

Upon motion it was voted that the thanks of the society be tendered to Drs. Briggs, Dorland, and Searles for presenting the memorial upon the death of Dr. Brooks, and to Dr. Hauenstein for presenting the memorial upon the death of Dr. Devening.

Upon motion of Dr. STORCK it was voted that the secretary be requested to send copies of the memorials to the families of the deceased.

Dr. ABBOTT moved the adoption of the resolution introduced in writing by him at the last meeting, that Sec. 3, Art. V. be amended to read, "No candidate shall be present when the question of his admission is determined by the society."

The amendment was adopted.

Dr. BRIGGS gave written notice that he would move, at the next meeting, an amendment to the by-laws providing for the appointment of a permanent committee on necrology.

Dr. ABBOTT presented in writing, to be moved for adoption at the next meeting, an amendment to Article II., adding after the word secretary the words "assistant secretary," and after the word treasurer the words "assistant treasurer."

Dr. ABBOTT reported that he had received from the Buffalo MEDICAL AND SURGICAL JOURNAL a bill for $18.00 for copies of the JOURNAL for the last nine years.

Upon motion it was voted that an order be drawn to pay the bill.

The society then adjourned.

